# The role of the support network in a vulnerable mother’s personal, emotional and professional life

**DOI:** 10.1590/1980-220X-REEUSP-2024-0271en

**Published:** 2025-07-28

**Authors:** Daniela Claudia Silva Fortes, Mara Regina Santos da Silva, Kateline Simone Gomes Fonseca, Stanik Michel Lima Pires, Denise Liliane Silva Cruz

**Affiliations:** 1 Universidade Federal do Rio Grande, Pós-Graduação em Enfermagem, Rio Grande, RS, Brazil.; 2 Ministério de Saúde e da Segurança Social de Cabo Verde, Praia, Cabo Verde.

**Keywords:** Hospitals, Maternity, Social Vulnerability, Employment, Self Care, Maternal Behavior

## Abstract

**Objective:**

To understand the impact of the support network on reconciling motherhood experienced in conditions of vulnerability in one’s personal, emotional and professional life.

**Method:**

This is a qualitative, exploratory and descriptive study carried out with 15 mothers living on the island of São Vicente, in Cape Verde, Africa. Data were collected in June and July 2022, through semi-structured interviews. For data analysis, a matrix was constructed based on the construct of maternal sensitivity proposed by Ainsworth, and the structure was inspired by discursive textual analysis.

**Results:**

The results are organized into three categories: the first addresses “Conciliation/conflict of the relationship between motherhood and emotional life”; the second addresses “Interdependence between motherhood and emotional life”; and the third illustrates “Prioritization of child care over mothers’ individuality”. The categories highlighted the difficulty of mothers in vulnerable conditions in reconciling motherhood and their personal, emotional and professional lives, as there is no separation of the roles they exercise, resulting in overlapping and prioritization of motherhood over other spheres.

**General considerations:**

The fragility of participants’ support network results in personal sacrifices for the sake of their children. A cohesive support network is needed, with whom they can share maternal responsibilities.

## INTRODUCTION

Motherhood is one of the experiences in a woman’s life cycle that requires changes and adaptations that allow her to find a balance between her routine prior to motherhood and the new condition with the arrival of children^(1)^. This is an experience that cannot be lived in isolation, as it requires reconciliation with other dimensions of a woman’s life, namely the personal, emotional and professional dimensions.

Even in favorable conditions, balancing multiple roles is a stressful and conflict-generating experience. This task can be even more difficult in a vulnerable situation, particularly when the mother has a low level of education, scarce economic and family resources, lacks access to daycare, and is unaware of or does not receive support from her social network^(2)^. In this study, we assume as a condition of vulnerability the coexistence of cumulative factors, such as low income, low level of education, single parenthood, underemployment/unemployment, precarious housing and being the home’s providers.

In the specific context of Cape Verde, an island country located off the northwest coast of Africa, where this study was conducted, mothers face similar challenges and adversities. A survey revealed that the responsibility for caring for and educating children has a matrifocal and single-parent profile, with women responsible for around 97% of childcare and 72% of household chores, in addition to other tasks such as supporting the family^(3)^. Furthermore, in the absence of or limited access to daycare, the burden on mothers can be even more overwhelming, significantly impacting other aspects of their lives, namely personal, emotional and professional^(2)^.

A systematic review of women’s experiences of social support during pregnancy showed that, in an adverse context, where factors such as single parenthood, poverty, low education, unemployment coexist, and when maternal tasks take precedence over others, the mother dedicates most of her time to caring for her children, forgetting other dimensions of life, such as personal, emotional and professional ones^(4)^. In difficult circumstances, such as mental illness, poor social, emotional and financial support, etc., access to a support network in which they feel supported in carrying out their role as a mother, without sacrificing other aspects of their life, is crucial to balancing personal, emotional and professional responsibilities with their duties as a maternal care provider.

Studies show that recognizing the challenges faced by mothers in vulnerable situations can help mobilize the support network, allowing them to share responsibilities and alleviate daily difficulties as well as exercising other roles without feeling guilty^(5,6)^. In this way, it is possible to minimize the overload experienced by mothers in balancing motherhood and their personal, emotional and professional lives, especially in vulnerable conditions.

However, in situations of vulnerability where the support network is absent or unsatisfactory, mothers are required to adapt and reassess their priorities. Studies indicate that demanding care, support and protection of children constitute the highest priority socioculturally required of mothers, supported by imposed standards or practices of how to be a good mother^(4,7)^. Thus, sacrificing one’s personal, emotional and professional life for the sake of motherhood can be seen as an acceptable practice, as long as the result is child well-being. The approach adopted in several studies seeks to understand how the reconciliation of motherhood and the spheres of mothers’ lives can impact child development and well-being. However, few studies focus on the mother to understand her experiences in reconciling these spheres^(5,6,8)^.

These findings support this research, which aims to understand the impact of the support network on reconciling motherhood experienced in conditions of vulnerability in one’s personal, emotional and professional life.

## METHOD

### Study Design

This is a qualitative, exploratory and descriptive study, structured according to the COnsolidated criteria for REporting Qualitative research (COREQ). The theory used is the concept of maternal sensitivity proposed by Mary Ainsworth^(9)^, with the aim of understanding the experience of motherhood in a context of vulnerability. According to the author, a sensitive mother is someone who can accurately interpret her children’s signals and respond in a congruent and immediate manner. This skill is developed through the relationship with children, which allows mothers to identify and satisfy the needs implicit in children’s behavior.

The choice of this framework is justified by the authors’ view of motherhood as a challenging experience in itself, in which sensitivity to adversity is essential for both mother and child. In other words, maternal sensitivity can function as a protective factor, promoting or reinforcing mothers’ ability to overcome challenges and satisfy their needs and those of their children.

### Place

The study was conducted with mothers living on the island of São Vicente, in the Republic of Cape Verde, in Africa. The population of Cape Verde is estimated at 579,598 inhabitants. In 2020, around 51.4% of the population benefited from at least one social support. According to UN Women and the United Nations Sustainable Development Programme in Cape Verde, women represent 46% of Cape Verdean families, with a poverty rate of 33%^(10,11)^.

The island of São Vicente is the second most populous island and was chosen as the study context because it is easier to geographically locate participants, in addition to the fact that the doctoral student who conducted the study resides on this island, which facilitated access to key informants from healthcare services who helped in selecting the mothers.

### Population and Selection Criteria

The study was conducted with 15 mothers. Participants were selected by convenience, with the help of professionals from primary healthcare services where they received prenatal care and care for their children during early childhood. In a private room and individually, mothers were invited to participate in the study. In accessible language, the concept of maternal sensitivity was explained to them, that is, what was considered to be “being a good mother”, referring to the roles of caregivers, protectors and providers of the basic physical and emotional needs of their children. Based on what was explained to them, they were asked to reflect on motherhood. Mothers who recognized themselves and declared themselves to be good mothers were included in the study, considering the vulnerable condition in which they lived.

Mothers over 18 years of age, with at least one child aged between 0 and 1 year, who declared themselves to be good mothers and met at least four of the cumulative vulnerability criteria, such as low income, low level of education, single parenthood, and underemployment/unemployment, precarious housing and being the home’s provider, were included.

### Data Collection

Data were collected in June and July 2022, through semi-structured interviews conducted at the mothers’ homes, with a day and time previously agreed upon and organized to ensure that daily routines and childcare were not altered. The interviews were conducted by the first author, lasted an average of 60 minutes, and were audio-recorded. Field notes were recorded after each interview. Data collection took place during the COVID-19 pandemic, and all safety protocols applied in the country were strictly followed.

To guide the interviews, a script was developed in accordance with the theoretical framework. The script is divided into six parts. The first part is dedicated to sociodemographic information, namely age, education, marital status, number of children, professional activity/occupation, family income, housing conditions, etc. The second part focuses on the perception of the role of mothers in vulnerable conditions, exploring questions such as: What do you consider to be problems in caring for your child(ren)? What do you do to manage these problems? What were the problems that affected you the most? What experiences in your role as a mother would you like to have been different?

The third part concerns the relationship between mother and child in terms of maternal responsibilities, with questions such as: How do you see yourself in your role as a mother? How do you think your child(ren) see you as a mother? How would you describe your relationship with your child(ren)? What influences your relationship with your child(ren) in a positive and negative way? The fourth part focused on the impact of motherhood in a vulnerable situation on mothers’ lives, emotional and professional projects, with questions such as: We would like you to reflect a little on how your role as a mother has impacted your professional, emotional and personal life. The fifth part focused on the temporal perspective, with a view to investigating the support network, both primary and secondary, and the type of support received since pregnancy. In this context, the following questions emerged: We would like you to reflect on your routine of caring for your child(ren) in the roles of caregiver, protector and provider of basic physical and emotional needs. When you needed help, who did you turn to? Where did you go? How often did you seek help?

Finally, the sixth part sought to understand the relationship between participants and primary healthcare services in São Vicente, Cape Verde. Questions such as: Do you use primary healthcare services in São Vicente? Describe the care you received in these healthcare services during and after pregnancy and childbirth. What actions did the professionals in these healthcare services take that helped you become a good mother?

### Data Analysis and Treatment

The material obtained in the interviews was analyzed and interpreted using a matrix constructed based on the theoretical elements of the concept of maternal sensitivity, organized according to the discursive textual analysis technique precepts proposed by Moraes and Galiazzi^(12)^, which allowed for a deep understanding and reconstruction of knowledge.

The analysis process consisted of four stages: 1) deconstruction and unitarization, in which, through text disintegration, units of analysis emerge; 2) categorization by grouping similar elements; 3) expression of new understandings (capture of the new emerging); and 4) an intuitive process of self-organized reconstruction that leads to the emergence of new understandings of the phenomenon.

### Ethical Aspects

Data were collected with the approval of two organizations – the National Data Protection Commission, with reference number 37/2022/CNPD, and the National Research Ethics Commission, with reference number 19/2022. Participants also signed the Informed Consent Form (ICF) before the start of the data collection process.

The data are associated with the Federal University of Rio Grande. All ethical principles described in Resolution 510/2016 were strictly respected. Participant privacy was respected, and they were identified by means of a code composed of the letter “M” followed by the order number in which the interviews were conducted.

## RESULTS

Fifteen mothers aged between 19 and 40 years participated in the study. Of these, 11 were in a romantic relationship with a partner. Participants had a precarious socioeconomic status, with five of them working and having a fixed family income of one minimum wage (13,000 Cape Verdean *escudos*) which, at the time of data collection, corresponded to US$ 126 and R$ 677.72. The remaining ten were unemployed and did not earn any income. Regarding education, four participants had incomplete elementary education, four had completed elementary education, five had incomplete secondary education and two had completed secondary education. Participants and their families lived in informal and insecure housing.

The data analysis generated three categories: the first is “Conciliation/conflict of the relationship between motherhood and emotional life”; the second is “Interdependence between motherhood and emotional life”; and the third is “Prioritization of child care over mothers’ individuality”.

### Conciliation/Conflict of the Relationship Between Motherhood and Emotional Life

This category included five mothers, who chose to continue working as a way of ensuring the support of their children (M3, M7, M8, M9, M12), and ten who left their jobs because they had no one to leave their children with (M1, M2, M4, M5, M6, M10, M11, M13, M14, M15). The relationship between motherhood and participants’ professional lives manifested itself in a complex dynamic, with them reporting that, in several situations, they felt divided.

*I have often thought about quitting my job to take care of my daughters, but if I do, how will I support them? It all depends on my salary.* (M8)

*Before, when I was working, I would leave the children at my sisters’ house. I spent most of my time on the streets, so I quit my job to take care of them... recently, my young children were left at home under the care of my 10-year-old daughter. When I came home from work, I found the baby injured, he had fallen, so I quit my job again to take care of them.* (M5)

The relationship between motherhood and the professional life of the participants who chose to leave their jobs to take care of their children is marked by demands from themselves and others. Their speech reveals that they are aware that one of the difficulties they face is the issue of supporting their children and that they have considered the possibility of returning to work. At the same time, they know that leaving their children alone at home and going to work can be considered neglect, which can jeopardize their custody of their children. Mothers revealed that the lack of a support network affects their professional life, making them more dependent on their family to meet their children’s needs.

*If I had someone to leave the children with, I could go back to work without worrying, because staying at home taking care of them and not having a job makes it difficult to even feed them.* (M1)

*When they are left alone, they can get hurt, so having a job and worrying about them being home alone is not worth it. They are children, I have to protect them.* (M5)

Employed mothers report that, in order to reconcile work and motherhood, they constantly seek solutions, such as leaving their children at home under the care of the oldest child, usually a teenager, or rekindling their relationship with their ex-partner. They stated that the solutions they found were the result of the pressure they faced to simultaneously fulfill their professional obligations and their duties as mothers, and that the support of their primary network was fundamental.

*My 15-year-old son stays with his brothers so I can work. I get home after midnight and my heart only rests when I see that they are well.* (M12)

M7 reported that, in order to work, she left her daughter in the care of family members, but that, on one of these occasions, the participant’s uncle sexually abused the girl.

*I left my daughter at a relative’s house to go to work. Her father was supposed to pick her up, and my uncle sexually abused her. I lost the confidence to leave her with someone and go to work. Now I am a mother of two girls...when I go to work, I feel like I have abandoned them.* (M7)

When examining the relationship between motherhood and participants’ professional lives, the influence of mothers’ support network becomes evident, as it is not always possible to reconcile these two dimensions. However, in both situations, the focus is on caring for children and responding to the maternal role.

### Interdependence Between Motherhood and Emotional Life

This category addresses the relationship between the exercise of motherhood and the emotional life of the mothers participating in the study, highlighting that, when they refer to this dimension of their lives, they limit themselves to the relationship with their partner. They explained that, in order to obtain support in caring for their children, they resort to their romantic relationship as part of their support network. Thus, they revealed that they started a new relationship, resumed or maintained an abusive relationship.

M3, M4, M7 and M13 stated that they had rekindled their relationship as a way of ensuring support in caring for their children. In these cases, the partner helped to share the care or provided financial support.

*I don’t allow my daughter’s stepfather to talk loudly to her, so we argue and as a result I’ve kicked him out of the house several times, but then he comes back. I need someone I trust to sleep with them so I can go to work*. (M7)

M1, M5, M6, M8, M9, M12 and M14 reported that they were subjected to an abusive relationship until they were able to organize themselves and take on the responsibility of supporting their children. They highlighted that, after the relationship ended, the children’s father stopped contributing to their care and support.

*When the relationship ended, he* [ex-partner] *stopped taking care of them and still threatens me... he attacked me, I got organized and got a job, I left home and, since then, I have been fighting alone for our children.* (M12)

*He* [ex-partner] *only helps if I have sex with him. If I refuse, he doesn’t give anything to the children and says that, because of me, he won’t help... and what happens to me, as a woman?* (M1)

M5 and M6 reported that they started a new relationship to be able to support and care for their children.

*I decided to stay single and take care of my children. Then I met my current partner. I was living in precarious conditions in a tin shack, but he told me he had a house with better conditions. I thought about them* [children] *and agreed to live with him* (M5)

Participants’ emotional lives were suggestive of being part of the support network used by them in responding to childcare, overriding their needs as women. The balance between these two spheres seems to be a strategy used by mothers, indicating that emotional life is a comprehensive part of motherhood and that the condition of vulnerability seems to have a significant impact on the decisions made.

### Prioritization of Child Care Over Mothers’ Individuality

When asked about motherhood in a vulnerable situation and their personal lives, participants’ discourses suggest that they have difficulty preserving their individuality, making motherhood the center of their lives. The arrival of children is seen as a moment that implies changes, revealing that they generally ignore their own needs in favor of those of their children.

*I don’t live for myself, I don’t even buy a pair of slippers, because I know I won’t have enough money to buy diapers... I have to save as much as possible and give up a lot of personal things, the focus is to guarantee my children’s food*. (M14)

When discussing motherhood and the impact of the vulnerable condition in which they find themselves, mothers emphasize the difficulties and experiences they are going through in the present, while also revealing concern for the future. M1, M2, M4, M5, M6, M7, M8, M11, M12 and M14 did not express their personal vision of how they see themselves in the future. Their response was limited to taking care of their children. The remaining participants demonstrated that they still dream of going back to school or finding a good job, although their priority continues to be ensuring a better future for their children.

*Honestly, my future involves taking care of my children. I don’t see any solutions for myself, nor do I have time for myself, and I take care of them alone.* (M1)

Ten participants (M4, M7, M8, M9, M10, M11, M12, M13, M14 and M15) reported that they became mothers as teenagers and that they had to reorganize themselves to move forward. In this situation, having or not having support was fundamental. Education is one of the aspects of personal life that was interrupted with the arrival of the child(ren).

*I got pregnant when I was 16. I had life plans like any teenager. I wanted to study and only then start a family... my mother threatened to throw me out of the house and my father supported me. I suffered from postpartum depression.* (M13)

Participants recognize the impact of motherhood on their personal lives, pointing to the arrival of children as a driving factor for change and adaptation. Their statements suggest that there is an overlap of tasks and that the needs of their children take precedence over their own, always being conditioned by the support network and conditions of vulnerability. They also add that motherhood takes precedence over self-care and self-fulfillment.

## DISCUSSION

The results of this study highlighted a variety of experiences of mothers in vulnerable conditions in the constant search for a balance between motherhood and their personal, emotional and professional lives. These are daily efforts with results and implications.

In relation to the relationship between motherhood in a vulnerable condition and professional life, a first observation allows us to infer the difficulty presented by participants in reconciling the two spheres. The results illustrate the fragility of the primary network and the omission and absence of the secondary network, resulting in increased financial stress for mothers who leave their jobs to care for their children.

Financial stress is often caused by the deprivation of basic goods for mothers and their children and generates feelings of anguish and despair due to the difficulty in ensuring sustenance^(8)^. This situation is in line with the results of this study, which found that everyday difficulties put pressure on these mothers who, without a source of income, generally have to look for an immediate solution that guarantees, at the very least, food for their children. These are mothers who already lived in vulnerable conditions and who, by giving up their jobs to be able to care for their children, increased their socioeconomic fragility.

The lack of support networks to help vulnerable mothers share the care of their children creates a deficit, and they feel responsible for filling it, resorting to solutions such as leaving their jobs. It is important to understand the underlying reasons that led these mothers to leave their jobs, as this not only compromises their financial independence, but also increases their vulnerability^(13)^. Participants are aware of the consequences of not having a job; however, they understand it as a protective measure for their children’s safety, since they left them alone at home so they could work.

This is a sensitive issue with two axes of analysis: if, on the one hand, quitting work jeopardizes the support of children, on the other hand, leaving them alone at home to go to work can be considered a negligent practice, which can jeopardize the custody of their children. In such cases, children may eventually be institutionalized, since rights that cannot be compromised are at stake, such as food, education, health and housing.

Studies show that when the support network is lacking, the mother is pressured by the context, in the search for means that allow her to safeguard the conditions for the protection, development and survival of her children, an action that should also be the responsibility of family members, public authorities and society^(14,15)^.

The results lead us to consider that, in a context of vulnerability, when a mother, due to lack of support from a support network, has to leave her job to take care of her children and, consequently, faces difficulties in meeting their basic needs, she should not be considered a negligent mother, but rather a neglected mother. Generally, what is assessed is the negative impact of socioeconomic deprivation resulting from unemployment on the cognitive, psychosocial, physical and educational development of children, without considering the mother as a subject of support and care^(16)^.

Mothers who managed to balance motherhood with their professional lives reported receiving support from their family network. However, this support is provided by older children or requires reconciling the marital relationship. This data helps us understand the resources that these mothers mobilize, showing that the absence or omission of the secondary network leaves them in a fragile situation, which may involve personal sacrifices, such as resuming the marital relationship or relying on older children to help care for the younger ones.

It is important to emphasize that children who support their mothers are also children or adolescents. Having children as a support network may seem paradoxical, since, on the one hand, their support is essential for mothers to be able to work, but, on the other hand, they take a responsibility that should fall to other spheres, such as the family, social support, programs and policies, and society.

Cape Verde is a country that has invested in safeguarding children’s rights, with actions to support families in vulnerable conditions, such as support for housing, food, education, health, among others^(17)^. Despite these investments, the data highlighted the daily challenges faced by mothers who reported not receiving support.

The existence of a supportive network is an essential resource for these mothers to be able to reconcile motherhood with their professional lives. When this support fails, it can increase the vulnerability they feel. Another point worthy of note is the fact that they all work in a canning factory, some on the night shift, which leads to the question of what kind of support exists for mothers in these situations.

Unfortunately, one of the participants who worked the night shift suffered sexual assault when she left her daughter at a relative’s house, which shows that the consequences of an inefficient support network can go beyond socioeconomic deprivation. Despite the importance of these findings, what predominates in the studies is the observation of the direct impact of the condition of vulnerability, leaving aside important issues such as the exposure of children to sexual violence, especially when they are away from their mother for a long time, who, out of necessity, had to go to work and entrusted the care of family members or acquaintances^(18)^.

Regarding the reconciliation of emotional life and motherhood, the results indicate that the former was often used as support for the latter. Mothers, as moderators between the context of vulnerability and their children, end up experiencing peculiar experiences, often involving personal sacrifices^(7)^. The results of this study show that, in order to ensure a support network in caring for their children, mothers can choose to remain in an abusive relationship, rekindle the relationship or start a new one, revealing the price they are willing to pay to safeguard their children’s well-being.

Although, in that context, this solution seemed ideal, in fact it is not, because with the adequate support of a support network, it is likely that they would not need to resort to their marital relationship to care for their children. Among the symbolisms surrounding motherhood, protecting children from adversity is a premise of validation as a good mother and, when they feel helpless, mothers usually mobilize the support network available to fulfill the maternal role of care and protection^(19)^.

Reestablishing a marital relationship in order to obtain the support of children’s father in caring for children also raises the issue of paternal responsibility. In the context of the study, it is common to find absent or negligent fathers, with the burden usually falling on mothers. The absence or omission of fathers implies an overload of care and finances for mothers who, in a context of vulnerability, may feel it is their duty to fill the gap left by assuming responsibility for children^(20)^. It can be assumed, then, that to alleviate the burden experienced, these participants found in the marital relationship a way to guarantee paternal responsibility and, thus, receive support in caring for their children.

These results allow us to affirm the need for practical support adapted to the circumstances of these mothers, in order to allow them to have greater control over their emotional life and preserve the boundary between motherhood and a romantic relationship. Motherhood and the pressure for a positive response to the maternal role often invade other spheres of a woman’s life, reducing her to the condition of mother, which can have consequences for her psychological well-being, in addition to the personal sacrifices involved^(21)^.

Maintaining an abusive relationship or submitting oneself to sexual violence by one’s partner or ex-partner as a way of ensuring food and housing for one’s children illustrates the complexity of the deficiency in the support and protection network of participants and highlights the importance of giving greater visibility to their experiences and sacrifices. These are issues that do not only affect mothers, as the negative impact of a violent home is also felt by children, who, when witnessing violence against their mother, become indirect victims, with a long-term impact on their life cycle^(18)^.

Although this study is limited in terms of the experience of violence against mothers and children, the phenomenon emerged spontaneously in the results, which led to the consideration that having to maintain an abusive relationship, reestablish or start another relationship as a means of support in caring for children is also a form of violence suffered by these women, as a result of the inefficiency of their support network. The support network provides protection to mothers in vulnerable situations, helping them to manage the adversities of daily life, reduce stress and be better able to have a warm and attentive relationship with their children, in addition to meeting their needs. Its absence or inefficiency generates the opposite, exposing them to more serious situations, such as violence^(22)^.

Concerning the motherhood and personal life axis, the results elucidated the overlap of motherhood in the other dimensions of these mothers’ lives, with their children as a priority, considering personal sacrifices to promote their well-being valid. Motherhood in a vulnerable condition, combined with the limitations of the context, social and personal pressure to respond positively, generally requires proactivity on the part of these mothers who, having no other way out, find themselves obliged to renounce their needs for the sake of their children, demonstrating their capacity for daily dedication and sacrifice^(23,24)^.

This ability is the result of the pressure that the condition of vulnerability imposes on motherhood. When they realize the failure of their support network, they sacrifice themselves for their children and, generally, they deny themselves. From the moment they become mothers in an adverse context, they take the responsibility of caring for and protecting their children from the difficulties of everyday life, going through exhausting experiences, generally involving successes and failures, with their choices having an impact on the healthy development of their children^(25)^. Although for them it may be understood as proof of maternal love, it is still an unfair solution, a consequence of the lack of support.

It is important to highlight that current results also shed light on the sacrifices faced by participants who became mothers in their teens, namely dropping out of school and entering the job market. This sacrifice could have been avoided if they had the support of a network that allowed them to reconcile their studies with motherhood and thus, in the long term, have a greater chance of breaking the cycle of vulnerability they experience.

The results support another study carried out with women in Cape Verde and allow us to argue that the annulment of personal and maternal identity in order to fulfill the maternal role in contexts of vulnerability and with few resources is also driven by cultural issues, in which mothers dedicate themselves so that their children do not have the same future as them^(26)^. Furthermore, the expectation of a positive response to the maternal role, with a focus on children’s healthy development, ends up putting pressure on these mothers who, without a cohesive support network, may seek at all costs to protect their children and, thus, avoid being classified as negligent mothers^(27)^.

It is believed that this study has the potential to raise awareness among the competent authorities, especially healthcare institutions, to review the approach adopted in the monitoring of women who are mothers in vulnerable conditions, including them as subjects of care so that they receive holistic attention that considers all aspects of their lives. Nursing has a privileged role in monitoring these mothers, from pregnancy to early childhood care. A review of the approach adopted and investments in research and education have the potential to produce better results in the medium and long term. The data supporting the study refer to one of the islands of Cape Verde and, despite local contributions, are not representative at a national level, so it is expected that other research can fill this gap.

## CONCLUSION

The results of this study show that the absence or inefficiency of a support network can interfere with a mother’s ability to balance motherhood with her personal, emotional and professional life, since motherhood often requires personal sacrifices, in which the priority is the well-being of her children over her own needs. There is usually a price to pay in terms of individuality, dreams and life projects.

It can be inferred that an insufficient or non-existent support network means that responsibility falls exclusively on the mother, leading her to exhaustion and helplessness. Failures in the support network can be the cause of complex experiences, namely socioeconomic deprivation, abusive relationships, reliance on the marital relationship as a support for care, children’s participation in the care of their siblings and personal sacrifices, among others.

Mothers can effectively balance their personal, emotional and professional lives with the help of an effective and robust support system, without resorting to drastic measures to fulfill their obligations. However, it is essential to recognize that the support network of participants in this study is not efficient, which suggests that social support organizations existing in the context of the study should include mothers as a focus of care and support, recognizing their experiences and offering practical support adapted to their circumstances.

## DATA AVAILABILITY

The data that support the results of this study are available in the publication itself.
