# Supplementary material for: The role of the support network in a vulnerable mother’s personal, emotional and professional life
Source: Rev Esc Enferm USP. 2025 Jul 28;59:e20240271. doi: 10.1590/1980-220X-REEUSP-2024-0271en (PMC12309522; doi:10.1590/1980-220X-REEUSP-2024-0271en)
Supplement: Translation

# Translation: O papel da rede de apoio na vida pessoal, afetiva e profissional damãe em vulnerabilidade

**DOI:** 10.1590/1980-220X-REEUSP-2024-0271pt

**Authors:** Daniela Claudia Silva Fortes, Mara Regina Santos da Silva, Kateline Simone Gomes Fonseca, Stanik Michel Lima Pires, Denise Liliane Silva Cruz

**Affiliations:** 1 Universidade Federal do Rio Grande, Pós-Graduação em Enfermagem, Rio Grande, RS, Brasil.; 2 Ministério de Saúde e da Segurança Social de Cabo Verde, Praia, Cabo Verde.

**Keywords:** Maternidade, Vulnerabilidade Social, Emprego, Autocuidado, Comportamento Materno

## Abstract

**Objetivo:**

Compreender o impacto da rede de apoio na conciliação da maternidade vivenciada em condições de vulnerabilidade em sua vida pessoal, afetiva e profissional.

**Método:**

Estudo qualitativo, exploratório descritivo, realizado com 15 mães residentes na ilha de São Vicente, em Cabo Verde, África. Os dados foram coletados em junho e julho de 2022, por meio de entrevistas semiestruturadas. Para a análise dos dados foi construída uma matriz a partir do constructo de sensibilidade materna proposto por Ainsworth e a estrutura foi inspirada na análise textual discursiva.

**Resultados:**

Os resultados estão organizados em três categorias: a primeira aborda a “Conciliação/conflito da relação maternidade e vida afetiva” a segunda “a interdependência entre maternidade e a vida afetiva” e o terceiro ilustra “A priorização do cuidado do filho em detrimento da individualidade da mãe”. As categorias evidenciaram a dificuldade das mães em condição de vulnerabilidade em conciliar a maternidade e a vida pessoal, afetiva e profissional, pois não há separação dos papéis por elas exercidos, resultando em sobreposição e priorização da maternidade em detrimento das outras esferas.

**Considerações gerais:**

A fragilidade da rede de apoio das participantes resulta em sacrifícios pessoais em prol dos filhos. Faz-se necessário uma rede de suporte coesa, com quem possam partilhar as responsabilidades maternas.

## INTRODUÇÃO

A maternidade é uma das experiências do ciclo vital de uma mulher que requer mudanças e adaptações que lhe permitam encontrar equilíbrio entre a rotina anterior à maternidade e a nova condição com a chegada dos filhos^(1)^. Trata-se de uma experiência que não pode ser vivida de forma isolada, uma vez que exige a sua conciliação com as outras dimensões da vida da mulher, nomeadamente as dimensões pessoal, afetiva e profissional.

Mesmo em condições favoráveis, conciliar múltiplos papéis é uma experiência geradora de estresse e conflitos. Essa tarefa pode ser ainda mais difícil em uma condição de vulnerabilidade, particularmente quando a mãe tem baixo grau de escolaridade, escassos recursos econômicos e familiares, falta de acesso a creches e desconhece ou não recebe suporte da rede social^(2)^. Neste estudo, assumimos como condição de vulnerabilidade a coexistência de fatores cumulativos, como a baixa renda, baixo nível de escolaridade, monoparentalidade, subemprego/desemprego, moradia precária e serem as provedoras do lar.

No contexto específico de Cabo Verde, país insular, localizado na costa noroeste da África, onde este estudo foi realizado, as mães enfrentam desafios e adversidades semelhantes. Um inquérito revelou que a responsabilidade de cuidar e educar tem perfil matrifocal e monoparental, cabendo às mulheres cerca de 97% dos cuidados com a prole e 72% dos cuidados domésticos, além de outras tarefas como sustentar a família^(3)^. Além disso, na ausência ou no acesso limitado às creches, o fardo para as mães pode ser ainda mais avassalador, repercutindo significativamente em outras dimensões da sua vida nomeadamente o pessoal, a afetiva e o profissional^(2)^.

Uma revisão sistemática sobre as experiências de apoio social das mulheres durante a gestação mostrou que, em contexto adverso, em que coexistem fatores como a monoparentalidade, pobreza, baixa escolaridade, desemprego, e quando as tarefas maternas se sobrepõem às demais, a mãe dedica a maior parte de seu tempo ao cuidado dos filhos, esquecendo-se de outras dimensões da vida, como a pessoal, a afetiva e a profissional^(4)^. Em circunstâncias difíceis, nomeadamente as doenças mentais, o fraco apoio social, emocional e financeiro, etc., o acesso a uma rede de suporte na qual se sinta apoiada no desempenho de seu papel de mãe, sem sacrificar outros aspetos da sua vida, é crucial para equilibrar as responsabilidades pessoais, afetivas e profissionais com os deveres como prestadora de cuidados maternos.

Estudos demonstram que o reconhecimento dos desafios enfrentados pelas mães em situações de vulnerabilidade pode ajudar a mobilizar a rede de suporte, permitindo partilhar responsabilidades e aliviar as dificuldades diárias, bem como o exercício dos outros papéis sem que se sentam culpada^(5,6)^. Deste modo, é possível minimizar a sobrecarga que é vivenciada pelas mães no equilíbrio entre a maternidade e a vida pessoal, afetiva e profissional, especialmente em condições de vulnerabilidade.

Entretanto, em situações de vulnerabilidade em que a rede de apoio é ausente ou insatisfatória, exigem que a mãe se adapte e reavalie as suas prioridades. Estudos indicam que a cobrança de cuidados, o apoio e a proteção dos filhos constituem a prioridade máxima exigida socioculturalmente à mãe, respaldada em padrões impostos ou práticas de como ser uma boa mãe^(4,7)^. Assim, sacrificar sua vida pessoal, afetiva e profissional em prol da maternidade pode ser vista como uma prática aceitável, desde que o resultado seja o bem-estar dos filhos. A abordagem adotada em diversos estudos procura compreender como a conciliação da maternidade e as esferas da vida da mãe podem impactar o desenvolvimento e o bem-estar da criança. No entanto, poucos estudos têm como foco a mãe para apreender suas vivências e experiências na conciliação dessas esferas^(5,6,8)^.

Estas constatações embasam esta investigação, que tem como objetivo compreender o impacto da rede de apoio na conciliação da maternidade vivenciada em condições de vulnerabilidade em sua vida pessoal, afetiva e profissional.

## MÉTODO

### Desenho do Estudo

Trata-se de um estudo qualitativo, do tipo exploratório e descritivo, estruturado de acordo com os Critérios Consensuais para a Publicação de Investigação Qualitativa (COREQ). A teoria utilizada é o conceito de sensibilidade materna proposto por Mary Ainsworth^(9)^, com o objetivo de compreender a experiência da maternidade num contexto de vulnerabilidade. Segundo a autora, uma mãe sensível é alguém que consegue interpretar com precisão os sinais de seu filho e responder de forma congruente e imediata. Esta competência é desenvolvida através da relação com a criança, a qual vai permitindo à mãe identificar e satisfazer as necessidades implícitas no seu comportamento.

A opção por esse referencial justifica-se pela visão dos autores acerca da maternidade como uma experiência desafiante por si só, em que a sensibilidade face às adversidades é fundamental tanto para a mãe como para a criança. Ou seja, a sensibilidade materna pode funcionar como um fator de proteção, promovendo ou reforçando a capacidade da mãe para superar os desafios e satisfazer as suas necessidades e as dos seus filhos.

### Local

O estudo foi realizado com mães residentes na ilha de São Vicente, na República de Cabo Verde, em África. A população de Cabo Verde é estimada em 579 598 habitantes. Em 2020, cerca de 51,4% da população beneficiava de, pelo menos, um apoio social. De acordo com a ONU Mulheres e o Programa das Nações Unidas para o Desenvolvimento Sustentável em Cabo Verde, as mulheres representam 46% das famílias caboverdianas, com uma taxa de pobreza de 33%^(10,11)^.

A ilha de São Vicente é a segunda ilha mais populosa e foi escolhida como contexto de estudo por ser mais fácil localizar geograficamente os participantes, além do fato de a doutoranda que conduziu o estudo residir nesta ilha, o que facilitou o acesso aos informantes-chave dos serviços de saúde que ajudaram na seleção das mães.

### População e Critério de Seleção

O estudo foi desenvolvido com 15 mães. As participantes foram selecionadas por conveniência, com a ajuda dos profissionais dos serviços de saúde de atenção primária onde realizaram o acompanhamento pré-natal e os cuidados com a criança durante a primeira infância. Em uma sala privativa e de forma individual, as mães foram convidadas a participar do estudo. Em uma linguagem acessível, foi-lhes explicado o conceito de sensibilidade materna, ou seja, o que estava a ser considerado como “ser boa mãe”, referindo-se aos papéis de cuidadora, protetora e provedora das necessidades básicas, físicas e emocionais dos filhos. A partir do que lhes foi explicado, foi-lhes pedido que refletissem sobre a maternidade. Foram integradas no estudo as mães que se reconheceram e autodeclararam-se boas mães, considerando a condição de vulnerabilidade em que viviam.

Os critérios de inclusão no estudo foram os seguintes: ter mais de 18 anos de idade; ter pelo menos um filho com idade entre zero e um ano; declarar-se boa mãe; e atender a pelo menos quatro dos critérios cumulativos de vulnerabilidade, nomeadamente: baixa renda, baixo nível de escolaridade, monoparentalidade, subemprego/desemprego, moradia precária e ser provedora do lar.

### Coleta de Dados

Os dados foram coletados durante os meses de junho e julho de 2022, mediante entrevistas semiestruturadas realizadas na residência das mães, com dia e hora previamente acordados e organizados de modo a garantir que as rotinas diárias e os cuidados prestados aos filhos não fossem alterados. As entrevistas foram conduzidas pela primeira autora, duraram, em média, 60 minutos e foram gravadas em áudio. Após cada entrevista, foram registadas notas de campo. A coleta de dados ocorreu durante a pandemia de COVID-19 e todos os protocolos de segurança aplicados no país foram rigorosamente cumpridos.

Para guiar as entrevistas, foi elaborado um roteiro de acordo com o referencial teórico. O roteiro está dividido em seis partes. A primeira parte é dedicada às informações sociodemográficas, nomeadamente a idade, escolaridade, estado civil, número de filhos, atividade profissional/ocupação, renda familiar, condições de moradia, etc. A segunda parte incide sobre a percepção do papel da mãe em condições vulneráveis, explorando questões como: o que você considera como sendo problema nos cuidados ao(s) filho(s)? O que você faz para administrar esses problemas? Quais foram os problemas que mais a marcaram? Que experiências no seu papel de mãe, você gostaria que tivessem sido diferentes?

A terceira parte é relativa à relação entre mãe e filho em termos de responsabilidades maternas, tendo sido feitas perguntas como: como se vê no seu papel de mãe? Como acha que o(s) seu(s) filho(s) a vêem como mãe? Como descreveria a relação com o(s) filho(s)? O que influencia a relação com o(s) filho(s) de forma favorável e de forma negativa? A quarta parte incidiu sobre o impacto da maternidade em situação de vulnerabilidade nos projetos de vida, afetivos e profissionais das mães, tendo sido colocadas questões como: “Gostaríamos que refletisse um pouco sobre como o seu papel de mãe tem repercutido na sua vida profissional, afetiva e pessoal”. A quinta parte incidiu sobre a perspectiva temporal, com vista a investigar a rede de apoio, tanto primária como secundária, e o tipo de apoio recebido desde a gravidez. Neste âmbito, foram colocadas questões como: “Gostaríamos que refletisse sobre a sua rotina de cuidados com o(s) filho(s) nas funções de cuidadora, protetora e provedora das necessidades básicas, físicas e emocionais.” Quando precisou de ajuda, a quem recorreu? Onde foi? Com que frequência recorreu a essa ajuda?

Por fim, a sexta parte procurou compreender a relação entre as participantes e os serviços de cuidados de saúde primários em São Vicente em Cabo Verde, tendo sido colocadas questões como: utiliza os serviços de atenção primária de saúde em São Vicente? Descreva o atendimento recebido nesses serviços de saúde durante e após a gravidez e o parto. Que ações desenvolvidas pelos profissionais nesses serviços de saúde contribuíram para se tornar uma boa mãe?

### Análise e Tratamento de Dados

O material obtido nas entrevistas foi analisado e interpretado por meio de uma matriz construída com base nos elementos teóricos do conceito de sensibilidade materna, organizada de acordo com os preceitos da técnica de análise textual-discursiva proposta por Moraes e Galiazzi^(12)^, a qual permitiu uma compreensão profunda e a reconstrução do conhecimento.

O processo de análise constou de quatro etapas: 1) desconstrução e unitarização, em que, por meio da desintegração dos textos, surgem unidades de análise; 2) categorização por agrupamento de elementos semelhantes; 3) expressão das novas compreensões (captação do novo emergente) e, finalmente, 4) um processo intuitivo de reconstrução auto-organizado que leva à emergência de novas compreensões do fenômeno.

### Aspectos Éticos

Os dados foram coletados com a aprovação de duas organizações - a Comissão Nacional de Proteção de Dados, com o número de referência 37/2022/CNPD, e o Comité Nacional de Ética em Pesquisa para a Saúde, com o número de referência 19/2022. As participantes também assinaram o Termo de Consentimento Livre e Esclarecido (TCLE) antes do início do processo de coleta de dados.

Os dados estão associados à Universidade Federal do Rio Grande-FURG. Todos os princípios éticos descritos na Resolução 510/2016 foram rigorosamente respeitados. A privacidade das participantes foi respeitada, tendo sido identificadas por meio de um código composto pela letra M seguida do número de ordem de realização das entrevistas.

## RESULTADOS

Participaram no estudo 15 mães com idades compreendidas entre 19 e 40 anos. Dessas, 11 mantinham uma relação afetiva com um companheiro. As participantes tinham um estatuto socioeconômico precário, sendo que cinco delas trabalhavam e possuíam renda familiar fixa no valor de um salário-mínimo (13.000 escudos cabo-verdianos), que, no momento da coleta dos dados, correspondia a 126 USD e R$ 677,72 reais. As dez restantes estavam desempregadas e não auferiam qualquer rendimento. Quanto à escolaridade, quatro participantes tinham o ensino fundamental incompleto, quatro o ensino fundamental completo, cinco o ensino médio incompleto e duas o ensino médio completo. As participantes e as respectivas famílias viviam em habitações informais e inseguras.

A análise dos dados gerou três categorias: a primeira intitulada “Conciliação/Conflito da relação maternidade e vida profissional”; a segunda, “A interdependência entre a maternidade e a vida afetiva”; e a terceira, “A priorização do cuidado do filho em detrimento da individualidade da mãe”.

### Conciliação/Conflito na Relação Maternidade e Vida Profissional

Nesta categoria foram incluídas as cinco mães que optaram por continuar no emprego como forma de garantir o sustento dos filhos (M3, M7, M8, M9, M12) e as dez que abandonaram o emprego por não terem com quem deixar os filhos (M1, M2, M4, M5, M6, M10, M11, M13, M14, M15). A relação entre a maternidade e a vida profissional das participantes manifestouse numa dinâmica complexa, com elas a referir que, em diversas situações, se sentiram divididas.

*Muitas vezes já pensei em abandonar o emprego para cuidar das minhas filhas, mas se o fizer como irei lhes sustentar, tudo depende do meu salário* (M8)

*Antes, para trabalhar, deixava as crianças na casa das minhas irmãs. Passava a maior parte do tempo na rua, por isso, abandonei o emprego para cuidar deles.... Recentemente, os meus filhos pequenos ficavam em casa sob os cuidados da minha filha de 10 anos. Quando cheguei do trabalho encontrei o bebê ferido, ele caiu, abandonei o emprego novamente para cuidar deles* (M5)

A relação entre a maternidade e a vida profissional das participantes que optaram por abandonar o emprego para cuidar dos filhos é marcada por cobranças próprias e de terceiros. O seu discurso revela que são conscientes de que uma das dificuldades enfrentadas é a questão do sustento dos filhos e que ponderaram a hipótese de regressar ao trabalho. Ao mesmo tempo, sabem que deixá-los sozinhos em casa e ir trabalhar pode ser considerado negligência, o que pode colocar em causa a guarda dos filhos. As mães revelaram que a falta de uma rede de suporte condiciona sua vida profissional, tornando-as mais dependentes da família para satisfazer as necessidades dos filhos.

*Se tivesse com quem deixar as crianças, poderia voltar a trabalhar sem preocupação, pois ficar em casa a cuidar deles e não ter emprego dificulta até a alimentação* (M1)

*Quando ficam sozinhos, podem magoar-se, portanto, ter um emprego e ficar preocupada com eles em casa sozinhos não compensa. São crianças, tenho de os proteger* (M5)

As mães empregadas referem que, para conciliar o emprego e a maternidade, procuram constantemente soluções, como, deixar os filhos em casa sob os cuidados do mais velho, geralmente um adolescente, ou reatar a relação com o ex-companheiro. Declararam que as soluções por elas encontradas são fruto da pressão que enfrentam para cumprir simultaneamente as suas obrigações profissionais e os seus deveres como mãe, e que o apoio da sua rede primária foi fundamental.

*O meu filho de 15 anos fica com os irmãos para eu poder trabalhar. Chego a casa depois da meia noite e só o meu coração só descansa quando vejo que estão bem* (M12)

A participante M7 relatou que, para trabalhar, deixava a filha aos cuidados de familiares, mas que, numa dessas vezes, o tio da participante abusou sexualmente da menina.

*Deixei a minha filha na casa de um familiar para ir trabalhar. O pai dela ficou de ir buscá-la, meu tio abusou sexualmente dela. Perdi a confiança para a deixar com alguém e ir trabalhar. Agora sou mãe de duas meninas...quando vou trabalhar, fico com a sensação de que as abandonei* (M7)

Ao examinar a relação entre a maternidade e a vida profissional das participantes, evidencia-se a influência da rede de apoio da mãe, que nem sempre consegue conciliar essas duas dimensões. Entretanto, em ambas as situações, o foco é o cuidado com os filhos e a resposta ao papel materno.

### A Interdependência da Maternidade e a Vida Afetiva

Esta categoria aborda a relação entre o exercício da maternidade e a vida afetiva das mães participantes no estudo, destacando que, quando se referem a esta dimensão de suas vidas, limitam-se à relação com o companheiro. Elucidaram que, para conseguirem apoio nos cuidados aos filhos, recorrem ao relacionamento amoroso como parte de sua rede de apoio. Assim, revelaram que iniciaram um novo relacionamento, reataram ou mantiveram uma relação abusiva.

As participantes M3, M4, M7 e M13 afirmaram ter reatado a relação como forma de garantirem apoio no cuidado dos filhos. Nesses casos, o companheiro ajuda a partilhar os cuidados ou fornece apoio financeiro.

*Não permito que o padrasto da minha filha fale alto com ela, pelo que discutimos e, em consequência, já o expulsei de casa várias vezes, mas depois ele regressa. Preciso que alguém de confiança durma com elas para eu ir trabalhar* (M7)

As participantes M1, M5, M6, M8, M9, M12 e M14 relataram que se submetem a um relacionamento abusivo até conseguirem organizar-se e assumir o sustento dos filhos. Salientaram que, com o término do relacionamento, o pai das crianças deixou de contribuir para os cuidados e no sustento delas.

*Com o término do relacionamento, ele* [ex-companheiro] *deixou de cuidar deles e ainda me ameaça.... Me agredia, fui me organizando e consegui um emprego, saí de casa e, desde então, venho lutando sozinha por nossos filhos* (M12)

*Ele* [ex-companheiro] *só ajuda se eu tiver relações sexuais com ele. Se me recusar, não dá nada aos filhos e diz que, por minha culpa, não vai ajudar..., E eu, como mulher, como fica?* (M1)

As participantes M5 e M6 referem que começaram um novo relacionamento para conseguirem dar suporte e cuidar dos filhos.

*Decidi ficar solteira e cuidar dos meus filhos. Depois, conheci o meu atual companheiro, eu vivia em condições precárias em um barraco de lata, mas disse-me que tinha uma casa com melhores condições. Pensei neles* [filhos] *e aceitei morar com ele* (M5)

A vida afetiva das participantes ficou sugestivo como parte da rede de suporte utilizada por elas na resposta ao cuidado dos filhos, sobrepondo as suas necessidades como mulher. O equilíbrio entre estas duas esferas parece ser uma estratégia utilizada pelas mães, indicando que a vida afetiva é parte integrante da maternidade e que a condição de vulnerabilidade parece ter um impacto significativo nas decisões tomadas.

### A Priorização do Cuidado do Filho em Detrimento da Individualidade da mãe

Quando questionadas sobre a maternidade em condição de vulnerabilidade e vida pessoal, o discurso das participantes sugere que têm dificuldade em preservar a sua individualidade, tornando a maternidade o centro de suas vidas. A chegada dos filhos é apontada como um momento que implica mudanças, revelando que, geralmente, ignoram suas necessidades em benefício das dos filhos.

*Não vivo para mim, nem compro um chinelo, porque sei que o dinheiro deixará falta para comprar fraldas... tenho de poupar o máximo possível e abrir mão de muita coisa pessoal, o foco é garantir a alimentação dos meus filhos* (M14)

Ao abordarem a maternidade e o impacto da condição de vulnerabilidade em que se encontram, as mães dão ênfase ás dificuldades e experiências que vivenciam no presente, revelando também preocupação com o futuro. As participantes M1, M2, M4, M5, M6, M7, M8, M11, M12 e M14 não manifestaram a sua visão pessoal sobre como se veem no futuro. A resposta limitava-se a cuidar dos filhos. As restantes participantes demonstram que ainda sonham com voltar a estudar ou conseguir um bom emprego, embora a prioridade continue a ser garantir um futuro melhor para os filhos.

*Sinceramente, o meu futuro passa por cuidar dos meus filhos. Não vejo soluções para mim, nem tenho tempo para mim, e cuido deles sozinha* (M1)

Dez participantes (M4, M7, M8, M9, M10, M11, M12, M13, M14 e M15) relataram que se tornaram mães na adolescência e que tiveram de se reorganizar para seguir em frente. Nessa situação, ter ou não ter apoio foi fundamental. A educação é um dos aspectos da vida pessoal que foi interrompida com a chegada do(s) filho(s).

*Engravidei aos 16 anos, tinha projetos de vida como qualquer adolescente, queria estudar e só depois constituir família... A minha mãe ameaçou pôr-me fora de casa e o meu pai apoiou-me. Sofri de depressão pós-parto* (M13)

As participantes reconhecem o impacto da maternidade em sua vida pessoal, apontando a chegada dos filhos como fator impulsionador para mudança e adaptação. As suas declarações sugerem que há sobreposição de tarefas e que as necessidades dos filhos se sobrepõem às suas, estando sempre condicionadas pela rede de apoio e pelas condições de vulnerabilidade. Acrescentam ainda que a maternidade se sobrepõe ao autocuidado e à autorrealização.

## DISCUSSÃO

Os resultados do estudo evidenciaram uma variedade de vivências das mães em condição de vulnerabilidade na busca constante de um equilíbrio entre a maternidade e a vida pessoal, afetiva e profissional. Trata-se de esforços diários com resultados e implicações.

Relativamente à relação entre a maternidade em condição de vulnerabilidade e a vida profissional, uma primeira observação permite inferir a dificuldade apresentada pelas participantes em conciliar as duas esferas. Os resultados ilustram a fragilidade na rede primária e a omissão e a ausência da rede secundária, tendo como consequência o aumento do estresse financeiro para as mães que abandonam o emprego para cuidar dos filhos.

O estresse financeiro é geralmente provocado pela privação de bens básicos para as mães e os seus filhos e gera sentimentos de angústia e desespero devido à dificuldade em garantir o sustento^(8)^. Tal conjuntura está de acordo com os resultados do presente estudo, que constatou que as dificuldades do cotidiano exercem pressão sobre essas mães que, sem fonte de rendimento, geralmente têm de procurar uma solução imediata que garanta, no mínimo, a alimentação dos filhos. São mães que já viviam em condição de vulnerabilidade e que, ao abandonarem o emprego para poder cuidar dos filhos, aumentaram sua fragilidade socioeconômica.

A falha na rede de apoio para ajudar as mães em situação de vulnerabilidade a partilhar os cuidados dos filhos gera um défice, e elas sentem-se na responsabilidade por o colmatar, recorrendo a soluções como o abandono do emprego. É importante compreender as razões subjacentes que levaram essas mães a deixar o emprego, pois, para além de comprometer a sua independência financeira, aumenta a sua vulnerabilidade^(13)^. As participantes têm consciência das consequências da falta do emprego, contudo, entendem-no como medida protetora da segurança dos filhos, uma vez que os deixavam sozinhos em casa para poderem trabalhar.

Esta é uma questão sensível com dois eixos de análise: se, por um lado, abandonar o emprego compromete o sustento dos filhos, por outro, deixá-los sozinhos em casa para ir trabalhar pode ser considerada uma prática negligente, o que pode colocar em causa a guarda dos filhos. Nesses casos, eventualmente as crianças podem ser institucionalizadas, dado que estão em causa direitos que não podem ser colocados em causa, como a alimentação, a educação, a saúde e a habitação.

Estudos apontam que, quando a rede de apoio é falha, a mãe é pressionada pelo contexto, na busca de meios que lhe permitam salvaguardar as condições para a proteção, o desenvolvimento e a sobrevivência dos filhos, uma ação que deveria ser também da responsabilidade de familiares, do poder público e da sociedade^(14,15)^.

Os resultados levam a ponderar que, em contexto de vulnerabilidade, quando uma mãe, por falta de suporte de uma rede de apoio, tem de abandonar o emprego para cuidar dos filhos e, por conseguinte, enfrenta dificuldades em satisfazer as suas necessidades básicas, não deve ser considerada uma mãe negligente, mas sim uma mãe negligenciada. Geralmente, o que se avalia é o impacto negativo da privação socioeconômica decorrente do desemprego no desenvolvimento cognitivo, psicossocial, físico e educacional das crianças, sem considerar a mãe como um sujeito de suporte e cuidado^(16)^.

As mães que conseguiram conciliar a maternidade com a vida profissional referiram receber suporte da rede familiar. No entanto, este apoio é prestado pelos filhos mais velhos ou exige a reconciliar da relação conjugal. Estes dados ajudam a compreender os recursos que essas mães mobilizam, evidenciando que a ausência ou a omissão da rede secundária as deixa numa situação de fragilidade, o que pode envolver sacrifícios pessoais, como o caso de reatar o relacionamento conjugal ou recorrer aos filhos mais velhos para ajudar a cuidar dos mais novos.

Convém frisar que os filhos que apoiam a mãe são também crianças ou adolescentes. Ter os filhos como rede de suporte pode parecer paradoxal, uma vez que, por um lado, o seu apoio é indispensável para que a mãe possa trabalhar, mas, por outro, assumem uma responsabilidade que deveria ser de outras esferas, como a família, o apoio social, os programas e as políticas e a sociedade.

Cabo Verde é um país que tem investido na salvaguarda dos direitos das crianças, com ações de suporte às famílias em condições de vulnerabilidade, como apoio a habitação, alimentação, educação, saúde, entre outros^(17)^. Não obstante estes investimentos, os dados evidenciaram os desafios diários enfrentados pelas mães que declararam não receber apoio.

A existência de uma rede de apoio resolutiva é um recurso indispensável para que estas mães consigam conciliar a maternidade com a vida profissional. Quando esse apoio falha, pode aumentar a vulnerabilidade sentida por elas. Outro ponto digno de atenção é o fato de que todas trabalharem numa fábrica de conservas, algumas na escala noturna, o que leva a questionar que tipo de apoio existe para mães nessas situações.

Infelizmente, uma das participantes que trabalhava no turno da noite, ao deixar a filha em casa de familiares, a criança sofreu agressão sexual, o que evidencia que as consequências da ineficiência da rede de apoio podem ir além da privação socioeconômica. Apesar da importância dessas descobertas, o que predomina nos estudos é a observação do impacto direto da condição de vulnerabilidade, deixando de parte questões importantes como a exposição das crianças à violência sexual, principalmente quando estão muito tempo longe da mãe que, por necessidade, teve de ir trabalhar e confiou os cuidados a familiares ou conhecidos^(18)^.

Quanto à conciliação da vida afetiva e da maternidade, os resultados indicam que o primeiro muitas vezes foi utilizado como suporte ao segundo. As mães, enquanto moderadoras entre o contexto de vulnerabilidade e os seus filhos, acabam por vivenciar experiências peculiares, muitas vezes envoltas em sacrifícios pessoais^(7)^. Os resultados deste estudo mostram que, para garantirem uma rede de apoio no cuidado dos filhos, as mães podem optar por manter uma relação abusiva, reatar o relacionamento ou iniciar um novo, revelando o preço que estão dispostas a pagar para salvaguardar o bem-estar dos filhos.

Embora, naquele contexto, essa solução parecesse ideal, na verdade não é, pois, com o suporte adequado de uma rede de apoio, é provável que não precisassem de recorrer ao relacionamento conjugal para cuidar dos filhos. Dos simbolismos que envolvem a maternidade, a proteção dos filhos da adversidade é uma premissa de validação como boa mãe e, quando se veem desamparadas, as mães geralmente mobilizam a rede de apoio à disposição para cumprir o papel materno de cuidado e proteção^(19)^.

Reatar o relacionamento conjugal para obter o apoio do pai das crianças no cuidado dos filhos levanta também aqui a questão da responsabilidade paterna. No contexto do estudo, é frequente encontrar pais ausentes ou omissos, ficando a sobrecarga geralmente a cargo da mãe. A ausência ou a omissão do pai implica uma sobrecarga de cuidados e financeira para a mãe, que, em contexto de vulnerabilidade, pode sentir-se no dever de colmatar a falta deixada, assumindo a responsabilidade pelos filhos^(20)^. Pode-se supor, então, que para aliviar a sobrecarga vivida, essas participantes encontraram no relacionamento conjugal uma forma de garantir uma responsabilidade paterna e, assim, receber apoio no cuidado dos filhos.

Estes resultados permitem afirmar a necessidade de apoio prático e adaptado às circunstâncias dessas mães, de modo a permitir-lhes ter maior domínio sobre a sua vida afetiva, e preservar o limite entre a maternidade e um relacionamento amoroso. A maternidade e a pressão por uma resposta positiva ao papel materno muitas vezes invadem as demais esferas de vida de uma mulher, reduzindo-a a condição de mãe, o que pode ter consequências no seu bem-estar psicológico, além dos sacrifícios pessoais envolvidos^(21)^.

Manter um relacionamento abusivo ou submeter-se à violência sexual por parte do companheiro ou ex-companheiro como forma de garantir a alimentação e a habitação para os filhos ilustra a complexidade da deficiência na rede de apoio e proteção das participantes e destaca a importância de dar maior visibilidade às suas experiências e sacrifícios. São questões que não afetam somente a mãe, pois o impacto negativo de um lar violento também é sentido pelos filhos, que, ao assistirem à violência contra a mãe, tornam-se vítimas indiretas, com impacto a longo prazo no seu ciclo vital^(18)^.

Embora o presente estudo seja limitado quanto à experiência da violência contra a mãe e os filhos, o fenômeno surgiu espontaneamente nos resultados, o que levou a considerar que ter de manter um relacionamento abusivo, reatar ou iniciar outro relacionamento como meio de apoio no cuidado dos filhos também é uma forma de violência sofrida por estas mulheres, em consequência da ineficiência de sua rede de apoio. A rede de apoio proporciona proteção às mães em situação de vulnerabilidade, ajudando-as a gerir as adversidades do cotidiano, a diminuir o estresse e a ter melhor condição para um relacionamento caloroso e atento com os filhos, além de suprir as suas necessidades. A sua ausência ou ineficiência gera o inverso, expondo-as a situações mais graves, como a violência^(22)^.

No que se refere ao eixo maternidade e vida pessoal, os resultados elucidaram a sobreposição da maternidade nas outras dimensões da vida dessas mães, tendo os filhos como prioridade, considerando válidos os sacrifícios pessoais para promover seu bem-estar. A maternidade em condição de vulnerabilidade, aliada às limitações do contexto, a pressão social e pessoal numa resposta positiva, geralmente requer proatividade por parte dessas mães que, não tendo outra saída, se veem na obrigação de renunciar às suas necessidades em prol dos filhos, demonstrando a sua capacidade de doação e sacrifício diários^(23,24)^.

Esta capacidade é fruto da pressão que a condição de vulnerabilidade impõe à maternidade. Ao aperceberem-se da falha na rede de apoio, sacrificam-se pelos filhos e, geralmente, anulam-se. A partir do momento em que se tornam mães em contexto adverso, assumem a responsabilidade de cuidar e proteger os filhos das dificuldades do cotidiano, passando por experiências exaustivas, geralmente envoltas em acerto e erros, com suas escolhas a repercutir-se no desenvolvimento saudável dos filhos^(25)^. Embora para elas possa ser entendido como prova do amor materno, não deixa de ser uma solução injusta, consequência da falta de apoio.

É importante salientar que os resultados atuais também elucidaram os sacrifícios enfrentados pelas participantes que se tornaram mães na adolescência, nomeadamente abandonar os estudos e entrar no mercado de trabalho. Este sacrifício poderia ser evitado se tivessem o apoio de uma rede que lhes permitisse conciliar os estudos com a maternidade e, assim, a longo prazo, ter maior probabilidade de interromper o círculo de vulnerabilidade que vivenciam.

Os resultados corroboram outro estudo realizado com mulheres em Cabo Verde e permitem argumentar que a anulação da identidade pessoal e materna para o cumprimento do papel materno em contextos de vulnerabilidade e com poucos recursos também é impulsionada por questões culturais, em que as mães se dedicam para que os filhos não tenham o mesmo futuro que elas^(26)^. Além disso, a expectativa de resposta positiva ao papel materno, com foco no desenvolvimento saudável do filho, acaba por ser uma pressão para essas mães que, sem uma rede de apoio coesa, podem procurar a todo custo proteger os filhos e, assim, evitar serem catalogadas como mães negligentes^(27)^.

Acredita-se que este estudo tem potencial para sensibilizar as entidades competentes, em especial as instituições de saúde, a rever a abordagem adotada no acompanhamento das mulheres que exerce a maternidade em condição de vulnerabilidade, incluindo-as como sujeitos de cuidados, de modo a receberem uma atenção holística que considere todos os aspectos das suas vidas. A Enfermagem tem um papel privilegiado no acompanhamento destas mães, desde a gestação até os cuidados na primeira infância. Uma revisão da abordagem adotada e investimentos em investigação e ensino têm potencial para produzir resultados melhores a médio e longo prazo. Os dados que sustentam o estudo referem-se a uma das ilhas de Cabo Verde e, apesar das contribuições locais, não são representativos a nível nacional, pelo que se espera que outras pesquisas possam colmatar esta lacuna.

## CONCLUSÃO

Os resultados deste estudo mostram que a ausência ou ineficiência da rede de apoio pode interferir na capacidade da mãe para equilibrar a maternidade com a sua vida pessoal, afetiva e profissional, uma vez que a maternidade exige, muitas vezes, sacrifícios pessoais, em que a prioridade é o bem-estar dos filhos em detrimento das suas próprias necessidades. Geralmente, há um preço a pagar, em termos de individualidade, sonhos e projetos de vida.

Pode-se inferir que uma rede de apoio insuficiente ou inexistente faz com que a responsabilidade recaia exclusivamente sobre a mãe, levando-a à exaustão e ao desamparo. As falhas na rede de apoio podem estar na origem de vivências complexas, nomeadamente a privação socioeconômica, os relacionamentos abusivos, o recurso ao relacionamento conjugal como suporte de cuidados, a participação dos filhos nos cuidados dos irmãos e os sacrifícios pessoais, entre outros.

As mães podem equilibrar eficazmente a sua vida pessoal, afetiva e profissional com a ajuda de um sistema de apoio eficaz e robusto, sem recorrer a medidas drásticas para cumprir as suas obrigações. No entanto, é fundamental reconhecer que a rede de apoio das participantes deste estudo não é eficiente, o que sugere que as organizações de apoio social existentes no contexto do estudo devem incluir a mãe como foco de cuidado e apoio, reconhecendo as suas vivências e ofertando apoio prático e adaptado às suas circunstâncias.

## DISPONIBILIDADE DE DADOS

Os dados que embasam os resultados deste estudo estão disponíveis na própria publicação.

# Translation: El papel de la red de apoyo en la vida personal, afectiva y profesional de las madres en situación de vulnerabilidad

**DOI:** 10.1590/1980-220X-REEUSP-2024-0271es

**Authors:** Daniela Claudia Silva Fortes, Mara Regina Santos da Silva, Kateline Simone Gomes Fonseca, Stanik Michel Lima Pires, Denise Liliane Silva Cruz

**Affiliations:** 1 Universidade Federal do Rio Grande, Pós-Graduação em Enfermagem, Rio Grande, RS, Brasil.; 2 Ministério de Saúde e da Segurança Social de Cabo Verde, Praia, Cabo Verde.

**Keywords:** Maternidades, Vulnerabilidad Social, Empleo, Autocuidado, Conducta Materna

## Abstract

**Objetivo:**

Comprender el impacto de la red de apoyo en la conciliación de la maternidad vivida en condiciones de vulnerabilidad en la vida personal, emocional y profesional.

**Método:**

Estudio cualitativo, exploratorio, descriptivo, realizado con 15 madres residentes en la isla de São Vicente, en Cabo Verde, África. Los datos fueron recolectados en junio y julio de 2022, mediante entrevistas semiestructuradas. Para el análisis de los datos se construyó una matriz basada en el constructo de sensibilidad materna propuesto por Ainsworth, y la estructura se inspiró en el análisis textual discursivo.

**Resultados:**

Los resultados se organizan en tres categorías: la primera aborda la “Conciliación/conflicto de la relación entre maternidad y vida afectiva”; el segundo aborda la “Interdependencia entre la maternidad y la vida emocional”; y el tercero ilustra la “Priorización del cuidado del niño por encima de la individualidad de la madre”. Las categorías resaltaron la dificultad que tienen las madres en condición de vulnerabilidad para conciliar la maternidad con su vida personal, afectiva y profesional, ya que no existe una separación de los roles que desempeñan, generando superposiciones y priorización de la maternidad en detrimento de otras esferas.

**Consideraciones generales:**

La fragilidad de la red de apoyo de los participantes resulta en sacrificios personales por el bien de sus hijos. Se necesita una red de apoyo cohesionada, con la que puedan compartir las responsabilidades maternas.

## INTRODUCCIÓN

La maternidad es una de las experiencias del ciclo vital de la mujer que requiere cambios y adaptaciones que le permitan encontrar el equilibrio entre la rutina anterior a la maternidad y la nueva condición con la llegada de los hijos^(1)^. Se trata de una experiencia que no puede vivirse de forma aislada, ya que exige su conciliación con otras dimensiones de la vida de la mujer, en particular las dimensiones personal, afectiva y profesional.

Conciliar múltiples roles, incluso en condiciones favorables, es una experiencia generadora de estrés y conflictos. Esta tarea puede ser aún más difícil en una situación de vulnerabilidad, especialmente cuando la madre tiene un bajo nivel educativo, escasos recursos económicos y familiares, falta de acceso a guarderías y desconoce o no recibe apoyo de la red social^(2)^. En este estudio, asumimos como condición de vulnerabilidad la coexistencia de factores acumulativos, como bajos ingresos, bajo nivel educativo, monoparentalidad, subempleo/desempleo, vivienda precaria y ser el sustento del hogar.

En el contexto específico de Cabo Verde, país insular situado en la costa noroeste de África, donde se realizó este estudio, las madres se enfrentan a retos y adversidades similares. Una encuesta reveló que la responsabilidad del cuidado y la educación tiene un perfil matrifocal y monoparental, correspondiendo a las mujeres alrededor del 97% del cuidado de los hijos y el 72% de las tareas domésticas, además de otras tareas como el sustento de la familia^(3)^. Además, en ausencia o con acceso limitado a guarderías, la carga para las madres puede ser aún más abrumadora, lo que repercute significativamente en otras dimensiones de su vida, en particular la personal, la afectiva y la profesional^(2)^.

Una revisión sistemática sobre las experiencias de apoyo social de las mujeres durante el embarazo mostró que, en un contexto adverso, en el que coexisten factores como la monoparentalidad, la pobreza, el bajo nivel educativo, el desempleo y cuando las tareas maternas se superponen a las demás, la madre dedica la mayor parte de su tiempo al cuidado de los hijos, olvidándose de otras dimensiones de la vida, como la personal, afectiva y profesional^(4)^. En circunstancias difíciles, como las enfermedades mentales, el escaso apoyo social, emocional y financiero, etc., el acceso a una red de apoyo en la que se sienta respaldada en el desempeño de su papel de madre, sin sacrificar otros aspectos de su vida, es crucial para equilibrar las responsabilidades personales, afectivas y profesionales con los deberes como cuidadora materna.

Los estudios demuestran que el reconocimiento de los retos a los que se enfrentan las madres en situaciones de vulnerabilidad puede ayudar a movilizar la red de apoyo, permitiendo compartir responsabilidades y aliviar las dificultades cotidianas, así como el ejercicio de otros roles sin sentirse culpables^(5,6)^. De este modo, es posible minimizar la sobrecarga que experimentan las madres al equilibrar la maternidad y la vida personal, afectiva y profesional, especialmente en condiciones de vulnerabilidad.

Sin embargo, en situaciones de vulnerabilidad en las que la red de apoyo es inexistente o insatisfactoria, se exige a la madre que se adapte y reevalúe sus prioridades. Los estudios indican que el cuidado, el apoyo y la protección de los hijos constituyen la máxima prioridad exigida socioculturalmente a la madre, respaldada por normas impuestas o prácticas sobre cómo ser una buena madre^(4,7)^. Así, sacrificar la vida personal, afectiva y profesional en pro de la maternidad puede considerarse una práctica aceptable, siempre que el resultado sea el bienestar de los hijos. El enfoque adoptado en diversos estudios trata de comprender cómo la conciliación de la maternidad y las esferas de la vida de la madre pueden repercutir en el desarrollo y el bienestar del niño. Sin embargo, pocos estudios se centran en la madre para comprender sus vivencias y experiencias en la conciliación de estas esferas^(5,6,8)^.

Estas constataciones fundamentan esta investigación, cuyo objetivo es comprender el impacto de la red de apoyo en la conciliación de la maternidad vivida en condiciones de vulnerabilidad en su vida personal, afectiva y profesional.

## MÉTODO

### Diseño del Estudio

Se trata de un estudio cualitativo, de tipo exploratorio y descriptivo, estructurado de acuerdo con los Criterios Consensuados para la Publicación de Investigación Cualitativa (COREQ). La teoría utilizada es el concepto de sensibilidad materna propuesto por Mary Ainsworth^(9)^, con el objetivo de comprender la experiencia de la maternidad en un contexto de vulnerabilidad. Según la autora, una madre sensible es aquella que es capaz de interpretar con precisión las señales de su hijo y responder de forma congruente e inmediata. Esta competencia se desarrolla a través de la relación con el niño, lo que permite a la madre identificar y satisfacer las necesidades implícitas en su comportamiento.

La elección de este marco de referencia se justifica por la visión de los autores sobre la maternidad como una experiencia desafiante en sí misma, en la que la sensibilidad ante las adversidades es fundamental tanto para la madre como para el niño. Es decir, la sensibilidad materna puede funcionar como un factor de protección, promoviendo o reforzando la capacidad de la madre para superar los desafíos y satisfacer sus necesidades y las de sus hijos.

### Lugar

El estudio se llevó a cabo con madres residentes en la isla de San Vicente, en la República de Cabo Verde, en África. La población de Cabo Verde se estima en 579 598 habitantes. En 2020, alrededor del 51,4% de la población se beneficiaba de al menos una prestación social. Según ONU Mujeres y el Programa de las Naciones Unidas para el Desarrollo Sostenible en Cabo Verde, las mujeres representan el 46% de las familias caboverdianas, con una tasa de pobreza del 33%^(10,11)^.

La isla de San Vicente es la segunda más poblada y fue elegida como contexto de estudio por ser más fácil localizar geográficamente a los participantes, además de que la doctoranda que realizó el estudio reside en esta isla, lo que facilitó el acceso a los informantes clave de los servicios de salud que ayudaron en la selección de las madres.

### Población y Criterios de Selección

El estudio se llevó a cabo con 15 madres. Las participantes fueron seleccionadas por conveniencia, con la ayuda de los profesionales de los servicios de atención primaria de salud donde realizaron el seguimiento prenatal y los cuidados del niño durante la primera infancia. En una sala privada y de forma individual, se invitó a las madres a participar en el estudio. En un lenguaje accesible, se les explicó el concepto de sensibilidad materna, es decir, lo que se consideraba «ser una buena madre», refiriéndose a las funciones de cuidadora, protectora y proveedora de las necesidades básicas, físicas y emocionales de los hijos. A partir de lo que se les explicó, se les pidió que reflexionaran sobre la maternidad. Se integraron en el estudio las madres que se reconocieron y se declararon buenas madres, teniendo en cuenta la situación de vulnerabilidad en la que vivían.

Los criterios de inclusión en el estudio fueron los siguientes: ser mayor de 18 años; tener al menos un hijo de entre cero y un año; declararse buena madre; y cumplir al menos cuatro de los criterios acumulativos de vulnerabilidad, a saber: bajos ingresos, bajo nivel educativo, monoparentalidad, subempleo/desempleo, vivienda precaria y ser la proveedora del hogar.

### Recolección de Datos

Los datos se recopilaron durante los meses de junio y julio de 2022, mediante entrevistas semiestructuradas realizadas en el domicilio de las madres, con fecha y hora previamente acordadas y organizadas de manera que no se alteraran las rutinas diarias y los cuidados prestados a los hijos. Las entrevistas fueron realizadas por la primera autora, duraron, en promedio, 60 minutos y fueron grabadas en audio. Después de cada entrevista, se registraron notas de campo. La recolección de datos se llevó a cabo durante la pandemia de COVID-19 y se cumplieron rigurosamente todos los protocolos de seguridad aplicados en el país.

Para orientar las entrevistas, se elaboró un guion de acuerdo con el marco teórico. El guion se divide en seis partes. La primera parte está dedicada a la información sociodemográfica, en particular la edad, el nivel de estudios, el estado civil, el número de hijos, la actividad profesional/ocupación, los ingresos familiares, las condiciones de vivienda, etc. La segunda parte se centra en la percepción del papel de la madre en condiciones vulnerables, explorando cuestiones como: ¿qué considera que es un problema en el cuidado de sus hijos? ¿Qué hace para gestionar estos problemas? ¿Cuáles han sido los problemas que más le han marcado? ¿Qué experiencias en su papel de madre le gustaría que hubieran sido diferentes?

La tercera parte se refiere a la relación entre madre e hijo en términos de responsabilidades maternas, con preguntas como: ¿Cómo se ve en su papel de madre? ¿Cómo cree que su(s) hijo(s) la ven como madre? ¿Cómo describiría la relación con su(s) hijo(s)? ¿Qué influye de manera favorable y negativa en la relación con su(s) hijo(s)? La cuarta parte se centró en el impacto de la maternidad en situaciones de vulnerabilidad en los proyectos vitales, afectivos y profesionales de las madres, con preguntas como: «Nos gustaría que reflexionara un poco sobre cómo su papel de madre ha repercutido en su vida profesional, afectiva y personal». La quinta parte se centró en la perspectiva temporal, con el fin de investigar la red de apoyo, tanto primaria como secundaria, y el tipo de apoyo recibido desde el embarazo. En este contexto, se plantearon preguntas como: «Nos gustaría que reflexionara sobre su rutina de cuidados con sus hijos en sus funciones de cuidadora, protectora y proveedora de las necesidades básicas, físicas y emocionales». Cuando necesitó ayuda, ¿a quién recurrió? ¿Dónde fue? ¿Con qué frecuencia recurrió a esa ayuda?

Por último, la sexta parte trató de comprender la relación entre las participantes y los servicios de atención primaria de salud en San Vicente, Cabo Verde, y se plantearon preguntas como: ¿utiliza los servicios de atención primaria de salud en San Vicente? Describa la atención recibida en estos servicios de salud durante y después del embarazo y el parto. ¿Qué acciones desarrolladas por los profesionales de estos servicios de salud contribuyeron a que se convirtiera en una buena madre?

### Análisis y Tratamiento de los Datos

El material obtenido en las entrevistas fue analizado e interpretado mediante una matriz construida a partir de los elementos teóricos del concepto de sensibilidad materna, organizada según los preceptos de la técnica de análisis textual-discursivo propuesta por Moraes y Galiazzi^(12)^, lo que permitió una comprensión profunda y la reconstrucción del conocimiento.

El proceso de análisis constó de cuatro etapas: 1) deconstrucción y unitarización, en la que, mediante la desintegración de los textos, surgen unidades de análisis; 2) categorización por agrupación de elementos similares; 3) expresión de las nuevas comprensiones (captura de lo nuevo emergente) y, finalmente, 4) un proceso intuitivo de reconstrucción autoorganizada que conduce a la emergencia de nuevas comprensiones del fenómeno.

### Aspectos Éticos

Los datos se recopilaron con la aprobación de dos organizaciones: la Comisión Nacional de Protección de Datos, con el número de referencia 37/2022/CNPD, y el Comité Nacional de Ética en Investigación para la Salud, con el número de referencia 19/2022. Las participantes también firmaron el Término de Consentimiento Libre y Esclarecido (TCLE) antes del inicio del proceso de recolección de datos.

Los datos están asociados a la Universidad Federal de Rio Grande-FURG. Se respetaron rigurosamente todos los principios éticos descritos en la Resolución 510/2016. Se respetó la privacidad de las participantes, que fueron identificadas mediante un código compuesto por la letra M seguida del número de orden de realización de las entrevistas.

## RESULTADOS

Participaron en el estudio 15 madres con edades comprendidas entre los 19 y los 40 años, de las cuales 11 mantenían una relación afectiva con una pareja. Las participantes tenían un estatus socioeconómico precario, cinco de ellas trabajaban y tenían ingresos familiares fijos equivalentes al salario mínimo (13 000 escudos caboverdianos), que, en el momento de la recolección de datos, correspondía a 126 dólares estadounidenses y 677,72 reales brasileños. Las diez restantes estaban desempleadas y no percibían ningún ingreso. En cuanto a la escolaridad, cuatro participantes no habían completado la enseñanza primaria, cuatro habían completado la enseñanza primaria, cinco no habían completado la enseñanza secundaria y dos habían completado la enseñanza secundaria. Las participantes y sus respectivas familias vivían en viviendas informales e inseguras.

El análisis de los datos generó tres categorías: la primera, titulada «Conciliación/conflicto entre la maternidad y la vida profesional»; la segunda, «La interdependencia entre la maternidad y la vida afectiva»; y la tercera, «La priorización del cuidado del hijo en detrimento de la individualidad de la madre».

### Conciliación/Conflicto Entre la Maternidad y la Vida Profesional

En esta categoría se incluyeron las cinco madres que optaron por continuar en su empleo como forma de garantizar el sustento de sus hijos (M3, M7, M8, M9, M12) y las diez que abandonaron su empleo por no tener con quién dejar a sus hijos (M1, M2, M4, M5, M6, M10, M11, M13, M14, M15). La relación entre la maternidad y la vida profesional de las participantes se manifestó en una dinámica compleja, en la que ellas mismas señalaron que, en diversas situaciones, se sentían divididas.

*Muchas veces he pensado en dejar el trabajo para cuidar de mis hijas, pero si lo hago, ¿cómo voy a mantenerlas? Todo depende de mi sueldo* (M8).

*Antes, para trabajar, dejaba a los niños en casa de mis hermanas. Pasaba la mayor parte del tiempo en la calle, por lo que dejé el trabajo para cuidar de ellos... Recientemente, mis hijos pequeños se quedaban en casa al cuidado de mi hija de 10 años. Cuando llegué del trabajo, encontré al bebé herido, se había caído, así que volví a dejar el trabajo para cuidar de ellos*(M5).

La relación entre la maternidad y la vida profesional de las participantes que optaron por abandonar el trabajo para cuidar de sus hijos está marcada por las exigencias propias y de terceros. Sus discursos revelan que son conscientes de que una de las dificultades a las que se enfrentan es la manutención de sus hijos y que han considerado la posibilidad de volver al trabajo. Al mismo tiempo, saben que dejarlos solos en casa e ir a trabajar puede considerarse negligencia, lo que puede poner en peligro la custodia de los hijos. Las madres revelaron que la falta de una red de apoyo condiciona su vida profesional, haciéndolas más dependientes de la familia para satisfacer las necesidades de sus hijos.

*Si tuviera a alguien con quien dejar a los niños, podría volver a trabajar sin preocupaciones, ya que quedarse en casa cuidándolos y no tener trabajo dificulta incluso la alimentación* (M1).

*Cuando se quedan solos, pueden hacerse daño, por lo que tener un trabajo y estar preocupada por ellos en casa solos no compensa. Son niños, tengo que protegerlos* (M5).

Las madres trabajadoras señalan que, para conciliar el trabajo y la maternidad, buscan constantemente soluciones, como dejar a los hijos en casa al cuidado del mayor, generalmente un adolescente, o reanudar la relación con su expareja. Declararon que las soluciones que encontraron son el resultado de la presión a la que se ven sometidas para cumplir simultáneamente con sus obligaciones profesionales y sus deberes como madres, y que el apoyo de su red primaria fue fundamental.

*Mi hijo de 15 años se queda con sus hermanos para que yo pueda trabajar. Llego a casa después de medianoche y solo mi corazón descansa cuando veo que están bien* (M12).

La participante M7 relató que, para poder trabajar, dejaba a su hija al cuidado de familiares, pero que, en una de esas ocasiones, el tío de la participante abusó sexualmente de la niña.

*Dejé a mi hija en casa de un familiar para ir a trabajar. Su padre se encargó de ir a recogerla, pero mi tío abusó sexualmente de ella. Perdí la confianza para dejarla con alguien y ir a trabajar. Ahora soy madre de dos niñas... cuando voy a trabajar, tengo la sensación de que las he abandonado* (M7)

Al examinar la relación entre la maternidad y la vida profesional de las participantes, se pone de manifiesto la influencia de la red de apoyo de la madre, que no siempre consigue conciliar estas dos dimensiones. Sin embargo, en ambas situaciones, el foco está en el cuidado de los hijos y en la respuesta al papel materno.

### La Interdependencia de la Maternidad y la Vida Afectiva

Esta categoría aborda la relación entre el ejercicio de la maternidad y la vida afectiva de las madres participantes en el estudio, destacando que, cuando se refieren a esta dimensión de sus vidas, se limitan a la relación con su pareja. Aclararon que, para conseguir apoyo en el cuidado de sus hijos, recurren a la relación amorosa como parte de su red de apoyo. Así, revelaron que habían iniciado una nueva relación, reanudado o mantenido una relación abusiva.

Las participantes M3, M4, M7 y M13 afirmaron haber reanudado la relación como forma de garantizar el apoyo en el cuidado de los hijos. En estos casos, la pareja ayuda a compartir los cuidados o proporciona apoyo económico.

*No permito que el padrastro de mi hija le levante la voz, por lo que discutimos y, como consecuencia, ya lo he echado de casa varias veces, pero luego vuelve. Necesito que alguien de confianza duerma con ellas para poder ir a trabajar* (M7).

Las participantes M1, M5, M6, M8, M9, M12 y M14 relataron que se someten a una relación abusiva hasta que consiguen organizarse y asumir el sustento de sus hijos. Destacaron que, al terminar la relación, el padre de los niños dejó de contribuir al cuidado y sustento de estos.

*Al terminar la relación, él* [excompañero] *dejó de cuidar de ellos y todavía me amenaza... Me agredía, me organicé y conseguí un trabajo, me fui de casa y, desde entonces, lucho sola por nuestros hijos* (M12)

*Él* [excompañero] *solo me ayuda si tengo relaciones sexuales con él. Si me niego, no les da nada a los niños y dice que, por mi culpa, no va a ayudar... Y yo, como mujer, ¿qué hago?* (M1)

Las participantes M5 y M6 mencionan que comenzaron una nueva relación para poder mantener y cuidar a sus hijos.

*Decidí quedarme soltera y cuidar de mis hijos. Después conocí a mi actual pareja, yo vivía en condiciones precarias en una chabola, pero él me dijo que tenía una casa en mejores condiciones. Pensé en ellos* [los hijos] *y acepté vivir con él* (M5).

La vida afectiva de las participantes se reveló como parte de la red de apoyo que utilizaban para cuidar de sus hijos, anteponiendo sus necesidades como mujeres. El equilibrio entre estas dos esferas parece ser una estrategia utilizada por las madres, lo que indica que la vida afectiva es parte integrante de la maternidad y que la condición de vulnerabilidad parece tener un impacto significativo en las decisiones que toman.

### La Priorización del Cuidado del Hijo en Detrimento de la Individualidad de la Madre

Cuando se les preguntó sobre la maternidad en condiciones de vulnerabilidad y la vida personal, el discurso de las participantes sugiere que tienen dificultades para preservar su individualidad, convirtiendo la maternidad en el centro de sus vidas. La llegada de los hijos se señala como un momento que implica cambios, revelando que, por lo general, ignoran sus necesidades en beneficio de las de sus hijos.

*No vivo para mí, ni me compro unas chancletas, porque sé que el dinero faltará para comprar pañales... tengo que ahorrar todo lo posible y renunciar a muchas cosas personales, el objetivo es garantizar la alimentación de mis hijos* (M14).

Al abordar la maternidad y el impacto de la situación de vulnerabilidad en la que se encuentran, las madres hacen hincapié en las dificultades y experiencias que viven en el presente, revelando también su preocupación por el futuro. Las participantes M1, M2, M4, M5, M6, M7, M8, M11, M12 y M14 no manifestaron su visión personal sobre cómo se ven en el futuro. La respuesta se limitaba a cuidar de sus hijos. El resto de participantes muestran que aún sueñan con volver a estudiar o conseguir un buen trabajo, aunque la prioridad sigue siendo garantizar un futuro mejor para sus hijos.

*Sinceramente, mi futuro pasa por cuidar de mis hijos. No veo soluciones para mí, ni tengo tiempo para mí, y los cuido sola* (M1).

Diez participantes (M4, M7, M8, M9, M10, M11, M12, M13, M14 y M15) relataron que se habían convertido en madres adolescentes y que habían tenido que reorganizarse para seguir adelante. En esa situación, contar o no con apoyo fue fundamental. La educación es uno de los aspectos de la vida personal que se vio interrumpido con la llegada de los hijos.

*Me quedé embarazada a los 16 años, tenía proyectos de vida como cualquier adolescente, quería estudiar y solo después formar una familia... Mi madre amenazó con echarme de casa y mi padre me apoyó. Sufrí depresión posparto* (M13).

Las participantes reconocen el impacto de la maternidad en su vida personal y señalan la llegada de los hijos como factor impulsor del cambio y la adaptación. Sus declaraciones sugieren que hay una superposición de tareas y que las necesidades de los hijos se superponen a las suyas, estando siempre condicionadas por la red de apoyo y las condiciones de vulnerabilidad. Añaden además que la maternidad se superpone al autocuidado y la autorrealización.

## DISCUSIÓN

Los resultados del estudio pusieron de manifiesto una variedad de experiencias de madres en situación de vulnerabilidad en la búsqueda constante de un equilibrio entre la maternidad y la vida personal, afectiva y profesional. Se trata de esfuerzos diarios con resultados e implicaciones.

En cuanto a la relación entre la maternidad en condiciones de vulnerabilidad y la vida profesional, una primera observación permite inferir la dificultad que presentan las participantes para conciliar ambas esferas. Los resultados ilustran la fragilidad de la red primaria y la omisión y ausencia de la red secundaria, lo que tiene como consecuencia el aumento del estrés financiero para las madres que abandonan el empleo para cuidar de sus hijos.

El estrés financiero suele estar provocado por la privación de bienes básicos para las madres y sus hijos y genera sentimientos de angustia y desesperación debido a la dificultad para garantizar el sustento^(8)^. Esta situación concuerda con los resultados del presente estudio, que constató que las dificultades cotidianas ejercen presión sobre estas madres que, sin fuente de ingresos, suelen tener que buscar una solución inmediata que garantice, como mínimo, la alimentación de sus hijos. Se trata de madres que ya vivían en condiciones de vulnerabilidad y que, al abandonar su empleo para poder cuidar de sus hijos, aumentaron su fragilidad socioeconómica.

La falta de una red de apoyo que ayude a las madres en situación de vulnerabilidad a compartir el cuidado de sus hijos genera un déficit, y ellas se sienten responsables de colmarlo, recurriendo a soluciones como el abandono del empleo. Es importante comprender las razones subyacentes que llevaron a estas madres a dejar su empleo, ya que, además de comprometer su independencia económica, aumenta su vulnerabilidad^(13)^. Las participantes son conscientes de las consecuencias de la falta de empleo, pero lo entienden como una medida para proteger la seguridad de sus hijos, ya que los dejaban solos en casa para poder trabajar.

Se trata de una cuestión delicada con dos ejes de análisis: si, por un lado, abandonar el empleo compromete el sustento de los hijos, por otro, dejarlos solos en casa para ir a trabajar puede considerarse una práctica negligente, lo que puede poner en peligro la custodia de los hijos. En estos casos, los niños pueden acabar siendo institucionalizados, ya que están en juego derechos que no pueden ponerse en tela de juicio, como la alimentación, la educación, la salud y la vivienda.

Los estudios señalan que, cuando la red de apoyo es deficiente, la madre se ve presionada por el contexto a buscar medios que le permitan salvaguardar las condiciones para la protección, el desarrollo y la supervivencia de sus hijos, una acción que también debería ser responsabilidad de los familiares, los poderes públicos y la sociedad^(14,15)^.

Los resultados llevan a considerar que, en un contexto de vulnerabilidad, cuando una madre, por falta de apoyo de una red de apoyo, tiene que abandonar su empleo para cuidar de sus hijos y, por lo tanto, enfrenta dificultades para satisfacer sus necesidades básicas, no debe ser considerada una madre negligente, sino una madre desatendida. Por lo general, se evalúa el impacto negativo de la privación socioeconómica derivada del desempleo en el desarrollo cognitivo, psicosocial, físico y educativo de los niños, sin considerar a la madre como sujeto de apoyo y cuidado^(16)^.

Las madres que lograron conciliar la maternidad con la vida profesional mencionaron que recibían apoyo de la red familiar. Sin embargo, este apoyo lo prestan los hijos mayores o exige la reconciliación de la relación conyugal. Estos datos ayudan a comprender los recursos que movilizan estas madres, poniendo de manifiesto que la ausencia o la omisión de la red secundaria las deja en una situación de fragilidad, lo que puede implicar sacrificios personales, como el caso de reanudar la relación conyugal o recurrir a los hijos mayores para que ayuden a cuidar de los más pequeños.

Cabe destacar que los hijos que apoyan a la madre también son niños o adolescentes. Contar con los hijos como red de apoyo puede parecer paradójico, ya que, por un lado, su apoyo es indispensable para que la madre pueda trabajar, pero, por otro, asumen una responsabilidad que debería recaer en otros ámbitos, como la familia, el apoyo social, los programas y las políticas y la sociedad.

Cabo Verde es un país que ha invertido en la salvaguarda de los derechos de los niños, con medidas de apoyo a las familias en situación de vulnerabilidad, como ayuda para la vivienda, la alimentación, la educación y la salud, entre otras^(17)^. A pesar de estas inversiones, los datos pusieron de manifiesto los retos a los que se enfrentan a diario las madres que declararon no recibir apoyo.

La existencia de una red de apoyo eficaz es un recurso indispensable para que estas madres puedan conciliar la maternidad con la vida profesional. Cuando este apoyo falla, puede aumentar la vulnerabilidad que sienten. Otro punto digno de atención es el hecho de que todas trabajan en una fábrica de conservas, algunas en el turno de noche, lo que lleva a preguntarse qué tipo de apoyo existe para las madres en estas situaciones.

Lamentablemente, una de las participantes que trabajaba en el turno de noche, al dejar a su hija en casa de familiares, la niña sufrió agresiones sexuales, lo que pone de manifiesto que las consecuencias de la ineficiencia de la red de apoyo pueden ir más allá de la privación socioeconómica. A pesar de la importancia de estos hallazgos, lo que predomina en los estudios es la observación del impacto directo de la condición de vulnerabilidad, dejando de lado cuestiones importantes como la exposición de los niños a la violencia sexual, sobre todo cuando pasan mucho tiempo lejos de la madre que, por necesidad, ha tenido que ir a trabajar y ha confiado su cuidado a familiares o conocidos^(18)^.

En cuanto a la conciliación de la vida afectiva y la maternidad, los resultados indican que la primera se utilizó a menudo como apoyo a la segunda. Las madres, como mediadoras entre el contexto de vulnerabilidad y sus hijos, acaban viviendo experiencias peculiares, a menudo envueltas en sacrificios personales^(7)^. Los resultados de este estudio muestran que, para garantizar una red de apoyo en el cuidado de los hijos, las madres pueden optar por mantener una relación abusiva, reanudar la relación o iniciar una nueva, lo que revela el precio que están dispuestas a pagar para salvaguardar el bienestar de sus hijos.

Aunque, en ese contexto, esta solución pareciera ideal, en realidad no lo es, ya que, con el apoyo adecuado de una red de apoyo, es probable que no necesitaran recurrir a la relación conyugal para cuidar de sus hijos. Entre los simbolismos que rodean la maternidad, la protección de los hijos frente a la adversidad es una premisa de validación como buena madre y, cuando se ven desamparadas, las madres suelen movilizar la red de apoyo a su disposición para cumplir el papel maternal de cuidado y protección^(19)^.

Reanudar la relación conyugal para obtener el apoyo del padre de los niños en el cuidado de estos también plantea aquí la cuestión de la responsabilidad paterna. En el contexto del estudio, es frecuente encontrar padres ausentes u omisos, quedando la carga generalmente a cargo de la madre. La ausencia u omisión del padre implica una sobrecarga de cuidados y financiera para la madre, que, en un contexto de vulnerabilidad, puede sentirse en la obligación de suplir la falta, asumiendo la responsabilidad de los hijos^(20)^. Cabe suponer, entonces, que para aliviar la sobrecarga que soportan, estas participantes encontraron en la relación conyugal una forma de garantizar la responsabilidad paterna y, así, recibir apoyo en el cuidado de los hijos.

Estos resultados permiten afirmar la necesidad de un apoyo práctico y adaptado a las circunstancias de estas madres, a fin de permitirles tener un mayor control sobre su vida afectiva y preservar la frontera entre la maternidad y una relación amorosa. La maternidad y la presión por una respuesta positiva al papel materno a menudo invaden las demás esferas de la vida de una mujer, reduciéndola a la condición de madre, lo que puede tener consecuencias en su bienestar psicológico, además de los sacrificios personales que ello implica^(21)^.

Mantener una relación abusiva o someterse a la violencia sexual por parte de la pareja o expareja como forma de garantizar la alimentación y la vivienda de los hijos ilustra la complejidad de las deficiencias en la red de apoyo y protección de las participantes y destaca la importancia de dar mayor visibilidad a sus experiencias y sacrificios. Son cuestiones que no solo afectan a la madre, ya que el impacto negativo de un hogar violento también lo sufren los hijos, que al presenciar la violencia contra la madre se convierten en víctimas indirectas, con un impacto a largo plazo en su ciclo vital^(18)^.

Aunque el presente estudio es limitado en cuanto a la experiencia de la violencia contra la madre y los hijos, el fenómeno surgió espontáneamente en los resultados, lo que llevó a considerar que tener que mantener una relación abusiva, reanudar o iniciar otra relación como medio de apoyo en el cuidado de los hijos también es una forma de violencia que sufren estas mujeres, como consecuencia de la ineficiencia de su red de apoyo. La red de apoyo proporciona protección a las madres en situación de vulnerabilidad, ayudándolas a gestionar las adversidades de la vida cotidiana, a reducir el estrés y a tener mejores condiciones para una relación cálida y atenta con sus hijos, además de satisfacer sus necesidades. Su ausencia o ineficiencia genera lo contrario, exponiéndolas a situaciones más graves, como la violencia^(22)^.

En lo que respecta al eje maternidad y vida personal, los resultados pusieron de manifiesto la superposición de la maternidad en las otras dimensiones de la vida de estas madres, que dan prioridad a sus hijos y consideran válidos los sacrificios personales para promover su bienestar. La maternidad en condiciones de vulnerabilidad, unida a las limitaciones del contexto y a la presión social y personal para dar una respuesta positiva, suele requerir proactividad por parte de estas madres que, al no tener otra salida, se ven obligadas a renunciar a sus necesidades en favor de sus hijos, demostrando su capacidad de entrega y sacrificio diario^(23,24)^.

Esta capacidad es el resultado de la presión que la condición de vulnerabilidad impone a la maternidad. Al darse cuenta de la falta de una red de apoyo, se sacrifican por sus hijos y, por lo general, se anulan a sí mismas. Desde el momento en que se convierten en madres en un contexto adverso, asumen la responsabilidad de cuidar y proteger a sus hijos de las dificultades cotidianas, pasando por experiencias agotadoras, generalmente envueltas en aciertos y errores, con sus elecciones repercutiendo en el desarrollo saludable de sus hijos^(25)^. Aunque para ellas pueda entenderse como una prueba de amor maternal, no deja de ser una solución injusta, consecuencia de la falta de apoyo.

Es importante destacar que los resultados actuales también han puesto de manifiesto los sacrificios a los que se enfrentan las participantes que se han convertido en madres adolescentes, en particular el abandono de los estudios y la incorporación al mercado laboral. Este sacrificio podría evitarse si contaran con el apoyo de una red que les permitiera conciliar los estudios con la maternidad y, así, a largo plazo, tener más probabilidades de romper el círculo de vulnerabilidad en el que viven.

Los resultados corroboran otro estudio realizado con mujeres en Cabo Verde y permiten argumentar que la anulación de la identidad personal y materna para cumplir con el papel de madre en contextos de vulnerabilidad y con pocos recursos también está impulsada por cuestiones culturales, en las que las madres se dedican a que sus hijos no tengan el mismo futuro que ellas^(26)^. Además, la expectativa de una respuesta positiva al papel materno, centrada en el desarrollo saludable del hijo, acaba ejerciendo presión sobre estas madres que, sin una red de apoyo cohesionada, pueden buscar a toda costa proteger a sus hijos y evitar así ser catalogadas como madres negligentes^(27)^.

Se cree que este estudio tiene el potencial de sensibilizar a las entidades competentes, en especial a las instituciones de salud, para que revisen el enfoque adoptado en el seguimiento de las mujeres que ejercen la maternidad en condiciones de vulnerabilidad, incluyéndolas como sujetos de cuidados, de modo que reciban una atención holística que tenga en cuenta todos los aspectos de sus vidas. La enfermería tiene un papel privilegiado en el seguimiento de estas madres, desde el embarazo hasta los cuidados en la primera infancia. Una revisión del enfoque adoptado y la inversión en investigación y enseñanza tienen el potencial de producir mejores resultados a medio y largo plazo. Los datos que sustentan el estudio se refieren a una de las islas de Cabo Verde y, a pesar de las contribuciones locales, no son representativos a nivel nacional, por lo que se espera que otras investigaciones puedan colmar esta laguna.

## CONCLUSIÓN

Los resultados de este estudio muestran que la ausencia o la ineficiencia de la red de apoyo puede interferir en la capacidad de la madre para conciliar la maternidad con su vida personal, afectiva y profesional, ya que la maternidad exige, a menudo, sacrificios personales, en los que la prioridad es el bienestar de los hijos en detrimento de sus propias necesidades. Por lo general, hay un precio que pagar en términos de individualidad, sueños y proyectos de vida.

Se puede inferir que una red de apoyo insuficiente o inexistente hace que la responsabilidad recaiga exclusivamente sobre la madre, lo que la lleva al agotamiento y al desamparo. Las deficiencias en la red de apoyo pueden ser el origen de experiencias complejas, como la privación socioeconómica, las relaciones abusivas, el recurso a la relación conyugal como apoyo para el cuidado, la participación de los hijos en el cuidado de los hermanos y los sacrificios personales, entre otros.

Las madres pueden equilibrar eficazmente su vida personal, afectiva y profesional con la ayuda de un sistema de apoyo eficaz y sólido, sin recurrir a medidas drásticas para cumplir con sus obligaciones. Sin embargo, es fundamental reconocer que la red de apoyo de las participantes en este estudio no es eficiente, lo que sugiere que las organizaciones de apoyo social existentes en el contexto del estudio deben incluir a la madre como foco de atención y apoyo, reconociendo sus experiencias y ofreciendo un apoyo práctico y adaptado a sus circunstancias.

## DISPONIBILIDAD DE DATOS

Los datos que respaldan los resultados de este estudio están disponibles en la propia publicación.
